# Partition refinement of WorldPop population spatial distribution data method: A case study of Zhuhai, China

**DOI:** 10.1371/journal.pone.0301127

**Published:** 2024-04-05

**Authors:** Rong Zhao, Shuang Wang, Yu Zhang, Chun Dong

**Affiliations:** 1 Chinese Academy of Surveying and Mapping, Beijing, China; 2 School of Geomatics, Liaoning Technical University, Fuxin, China; University of Strathclyde, UNITED KINGDOM

## Abstract

Currently, the core idea of the refined method of population spatial distribution is to establish a correlation between the population and auxiliary data at the administrative-unit level and, then, refine it to the grid unit. However, this method ignores the advantages of public population spatial distribution data. Given these problems, this study proposed a partition strategy using the natural break method at the grid-unit level, which adopts the population density to constrain the land class weight and redistributes the population under the dual constraints of land class and area weights. Accordingly, we used the dasymetric method to refine the population distribution data. The study established a partition model for public population spatial distribution data and auxiliary data at the grid-unit level and, then, refined it to smaller grid units. This method effectively utilizes the public population spatial distribution data and solves the problem of the dataset being not sufficiently accurate to describe small-scale regions and low resolutions. Taking the public WorldPop population spatial distribution dataset as an example, the results indicate that the proposed method has higher accuracy than other public datasets and can also describe the actual spatial distribution characteristics of the population accurately and intuitively. Simultaneously, this provides a new concept for research on population spatial distribution refinement methods.

## Introduction

Demographic data have broad application prospects in urban regional planning [1], resource allocation [2], disaster relief and assessment [3] below public health [4] and other research fields. Traditional demographic data use administrative divisions as the basic unit for showing a regional population, and expressing the distribution characteristics of a population from a spatial perspective is difficult. Although the spatial distribution data of the population solves the problem of spatial expression, the data scale within a small area does not adequately present the characteristics of the regional population distribution. Therefore, further research is necessary to improve the resolution and accuracy of population spatial distribution data on a fine scale within a small area.

Currently, the refinement method for population spatial distribution data is primarily based on the improvement of auxiliary data and models based on the population spatialization method. In early studies of population spatialization, several scholars considered land use data type as an influencing factor affecting the spatial distribution of the population. Following the assumption that the population density of the same land-use type was the same, a multiple regression relationship between population density and the area occupied by the corresponding land-use type was constructed, and the land-use type method was developed [5]. The principle of this method is simple and highly accurate. This method was employed to draw China’s 1995, 2000, and 2003 km grid data sets [6]. However, the influence of other factors on the spatial distribution of the populations was not considered. In addition to land-use types, factors such as road network, elevation, slope, housing construction, night light, and Point of Interest (POI) data also impact the spatial distribution [7]. The spatial distribution factor was established by fusing multi-source data, which reflects the spatial distribution of the population more accurately than single data. Dond et al. [8], Zhang et al. [9], and Mao et al. [10] investigated the spatial population distribution and geographical elements, obtained the weight coefficient of the grid population distribution, and distributed the population of the grid. Yong et al. [11] and Peng et al. [12] used geographic and multi-source information fusion data to achieve refined population allocation and simulation. Although the use of multi-source data increases the accuracy of the population spatial distribution data, the process of determining the weight value of the population spatial distribution is relatively complicated and difficult. Existing research adopts two main methods for determining the weight—namely, the multiple linear regression method [13] and machine learning [14]. Multivariate linear regression assumes that the relationship between the population and population spatial distribution impact factors is linear; however, the two are better reflected in complex nonlinear relationships [15]. The idea of the machine learning model is similar to that of a multivariate linear regression model. Population and auxiliary data are used as samples for the machine learning model, and the model relationship between them is established to obtain the influence weight value of auxiliary data on the population. The random forest model, neural networks, decision trees, and deep learning are widely used in population spatialization. The random forest model can not only obtain the weight value of each impact factor but also better simulate the complex nonlinear relationship between population and population impact factors, which have been widely used in recent years [16].

Research on refined population spatial distribution modeling methods predominantly improves model accuracy and multi-source auxiliary data, and the public population spatial distribution data are only used as comparative data to verify the accuracy of the population modeling method, which results in the public population spatial distribution dataset not being well-utilized. Representative public population data sets include the Gridded Population of the World (GPW), which is based on the area weight method [17]; the China km Grid Population Distribution Dataset that extracted land-use types, nighttime light intensity, and residential density to construct Chinese population spatial distribution weights [18]; the Global Population Dynamic Statistical Analysis Database of the weight model that was constructed using land use, road, and urban and village residential data (LandScan) [1, 19]; and the WorldPop data set that is fused by multi-source data, such as night light, land use, and road network information based on the random forest model [20, 21]. In the public population spatial distribution data set, the spatial resolution is mostly 1 km, and its spatial resolution is so low that meeting the fine research of population is difficult.

Previous studies found that WorldPop has the highest overall accuracy and spatial resolution in the public population spatial distribution dataset [10, 22]. However, the land cover data reflect the natural attributes of land rather than the social and economic attributes of land generated by human activities combined with land. Moreover, the land cover data with a spatial resolution of 30 m—as used in WorldPop—has a lower resolution. The above reasons precipitate inconsistencies between the spatial distribution of the population and the distribution of land use in small-scale population spatial analysis [23]. In addition, in the study of population refinement, the partition density method is typically used to model the population in the administrative division unit and, then, distribute the data to the grid unit, which ignores the error caused by the large span of the spatial scale in the cross-scale modeling and breaks the spatial autocorrelation of the population [24]. To solve this problem, the current study used land-use data to solve the problem of inconsistency between population spatial distribution and land-use distribution. Simultaneously, the relationship between population and auxiliary data is constructed at the grid-unit level to mitigate the problems caused by cross-scale [25, 26].

Therefore, this study proposes a partition refinement method for public WorldPop population spatial distribution data based on land-use data at the grid-unit level. Taking Zhuhai City, Guangdong Province, China, as an example, WorldPop data with a spatial resolution of 100 m were refined using land-use data with an accuracy better than 1 m considered as auxiliary data. Based on the partition method, the population was divided according to density, and the land-type weight of each population density area was calculated to reduce the heterogeneity of the population in spatial distribution. The original grid population of WorldPop was redistributed under the dual constraints of land-type weight and land-type area weight and refined to 25 m × 25 m population grid data. The accuracy and effectiveness of the fine population grid dataset obtained using this method were tested using demographic data of administrative units such as Township, Town, and Sub-district. Compared to previous research [11, 25, 27] on the refinement of population spatial distribution data, this method establishes a partition model at the grid-unit level, effectively utilizes public population spatial distribution data, and overcomes the dependence on demographic data. Further, our study solves the problem of the public population spatial distribution dataset being not sufficiently accurate to describe small-scale regions and low resolutions. This method offers the advantages of high operability, easy implementation, and easy data access and can also fulfill the requirements of analysis, management, and scientific research owing to its spatial accuracy.

## Study area and data

### Study area

**Fig 1:**
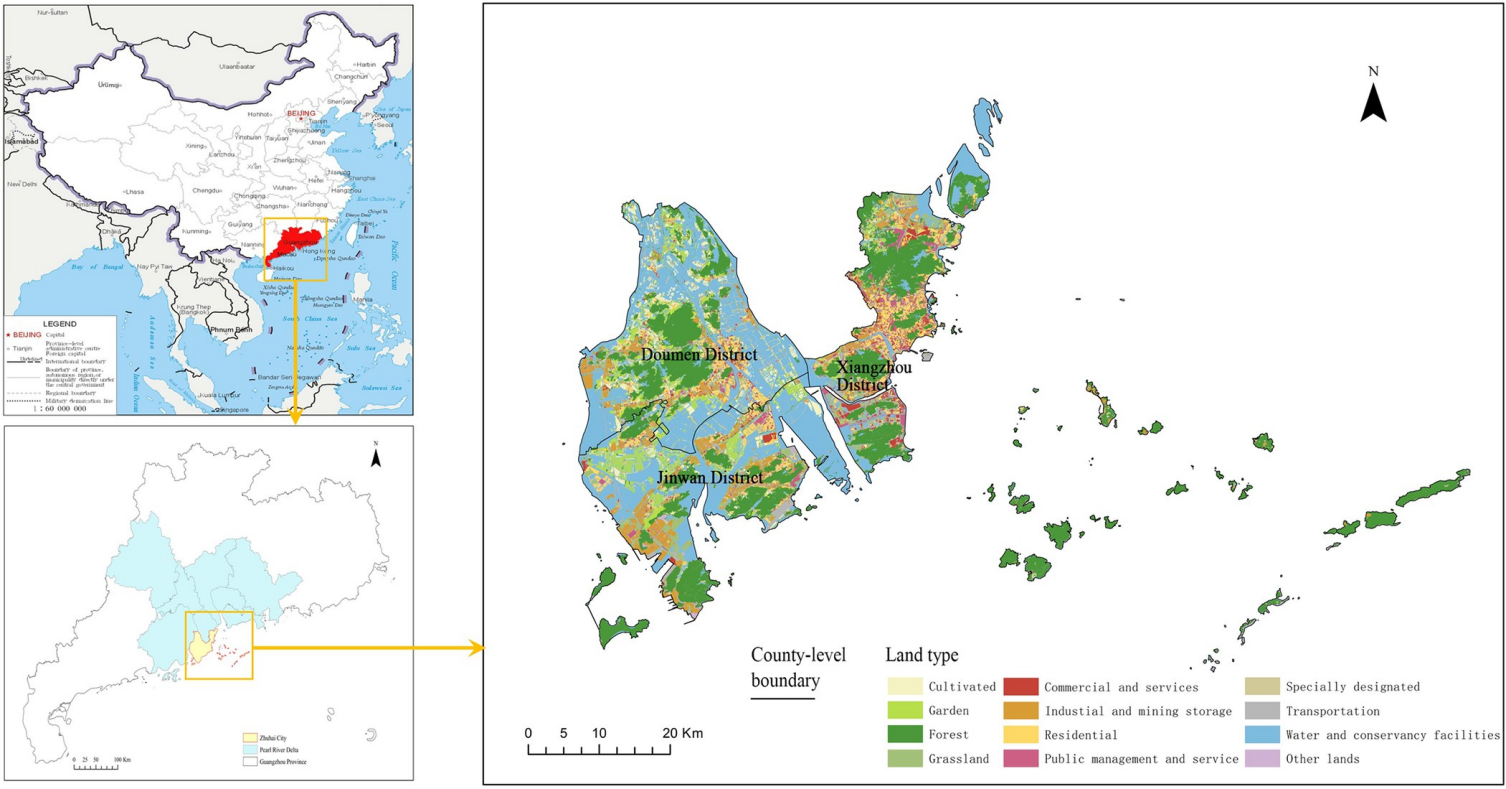
Zhuhai City geographical location map.

Zhuhai City, Guangdong Province, located in southern China was selected as the study area (Fig 1). It is a prefecture-level, central city in the Pearl River Delta, and an important node city in the Guangdong–Hong Kong–Macao Greater Bay Area. It faces Hong Kong across the sea in the east and Macau in the south. The city is under the jurisdiction of Xiangzhou, Doumen, and Jinwan, which are county-level administrative regions. According to the unified accounting of the Guangdong Provincial Bureau of Statistics, Zhuhai City’s regional gross domestic product (GDP) was CNY 348.194 billion in 2020, and the three administrative regions of Xiangzhou, Jinwan, and Doumen achieved regional GDP of CNY 234.753 billion, CNY 700.40 billion, and CNY 434.01 billion, respectively. The population of Zhuhai City is 2,439,585, with an average population density of 1411 people/km^2^. The population densities of Xiangzhou, Jinwan, and Doumen Districts are 2503 people/km^2^, 791 people/km^2^, and 995 people/km^2^, respectively.

As an important city in China’s special economic zone, Zhuhai City has a high level of urbanization development, and the population urbanization rate has reached 90.47%. Zhuhai City is an important port city in China with 10 national first-class ports. It has the largest sea area, largest islands, and longest coastline in the Pearl River Delta. The connectivity of the population space between local areas in Zhuhai City is interrupted, and the population is concentrated in urban areas. The population density between urban and rural areas is considerably different, and the spatial distribution of the population exhibits spatial heterogeneity. Therefore, conducting population spatial distribution refinement using Zhuhai City as the research area is challenging.

### Data sources

Details of the research data used in this study are listed in Table 1. Land-use data were used to calculate the land type and area weights. WorldPop data were used as the initial values before the population grid allocation. GPW and LandScan data were used for accuracy verification and comparison. Demographic data were compared to verify the effectiveness of the method.

**Table 1:** Datasets and sources.

Datasets	Format	Time	Source	Description
Land-use data	Vector	2020	http://gk.mnr.gov.cn/ysqgk/201905/t20190515_2411660.html	First-type land types, precision is better than 1 m.
Administrative division data	Vector	2020	http://gk.mnr.gov.cn/ysqgk/201905/t20190515_2411660.html	Zhuhai City,
				Prefectural Level, County-level, and Township-level Administrative Region
WorldPop	Grid	2020	https://hub.worldpop.org/	100 m spatial resolution
GPW	Grid	2020	https://sedac.ciesin.columbia.edu/	1 km spatial resolution
LandScan	Grid	2020	https://landscan.ornl.gov/	1 km spatial resolution
Population	Table	2020	http://tjj.zhuhai.gov.cn/	The Seventh National Population Census Township-level administrative region population data

#### Land-use data

Land-use data were obtained from the 2020 Land-Use Change Survey database and applied to the Ministry of Natural Resources of the People’s Republic of China. For 2020, the database was used to collect the latest remote sensing image data for the country. Data with a resolution better than 1 m were used in rural areas, and those with a resolution better than 0.2 m were used in urban villages to create a digital orthophoto map. Internal interpretation extracts information on land use changes, such as construction and agricultural land [28]. As illustrated in Fig 1, the first class of land-use data included cultivated, garden, forest, grass, commercial and service, industrial and mining storage, residential, public management and service, specially designated, transportation, water area and water conservancy facilities, and other land types.

#### Administrative division data

Administrative division data were obtained from the 2020 Land Change Survey database and applied to the Ministry of Natural Resources of the People’s Republic of China. China’s administrative regions are divided into four categories. The first to fourth levels are the provincial, prefectural, county-level, and township-level administrative regions, respectively. County-level administrative regions include categories such as counties, municipalities, cities, and municipal districts, and township-level administrative regions include townships, towns, and sub-districts.

#### WorldPop

The WorldPop project—established in 2013—combines AfriPop, AsiaPop, and AmeriPop population mapping projects to produce high-resolution population distribution data for low- and middle-income countries. The project aims to provide open population spatial distribution data for Africa, Asia, and Central and South America. Worldpop widely uses remote sensing and geospatial data sets, such as nighttime light, transportation network, land cover, and other data, to construct an asymmetric weight model; thereafter, it uses the random forest model to generate a grid prediction of population density at 100 m and 1 km spatial resolutions. Owing to its high spatial resolution and accuracy, this dataset is often used in comparative experiments on the spatialization of various types of population data to evaluate the effectiveness of other experiments. Some studies directly use the dataset as real population data inside the grid for model training [12].

#### Population

Population data were obtained from the seventh National Population Census, which is available from the Zhuhai City Bureau of Statistics [29]. The national census is a comprehensive investigation and registration of China’s existing population—generally conducted household-by-household and person-by-person. To ensure the authenticity, accuracy, and integrity of the data, the current study mainly aimed to comprehensively determine the number, structure, and distribution of the Chinese population—the most basic and important national data. The census focuses on mastering the analysis and predicting the development and changes in the existing population in various locations.

## Modeling and methodology

**Fig 2:**
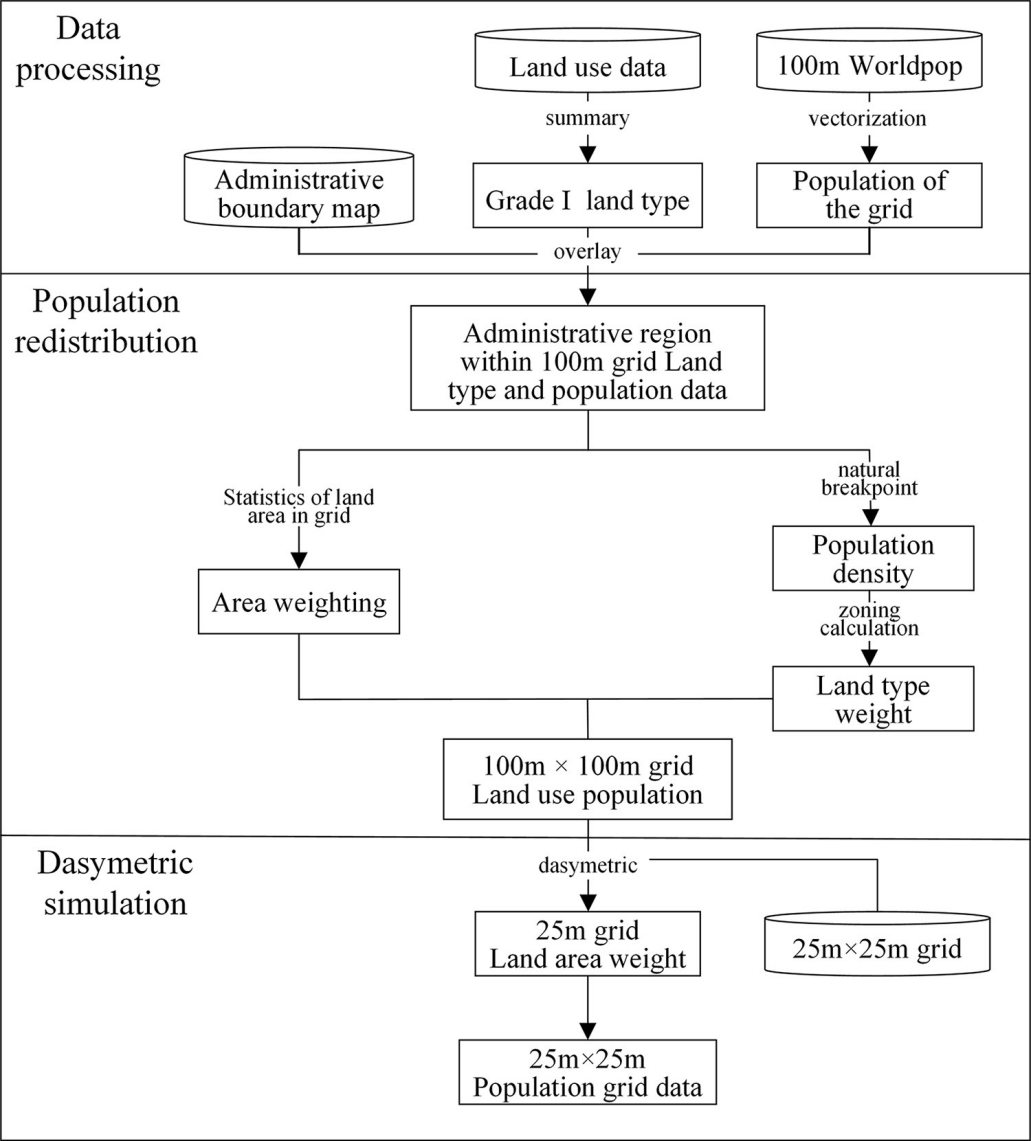
Workflow of the population grid data fine division method.

The workflow of the proposed method is presented in Fig 2. This method comprises three main stages—namely, data pre-processing, WorldPop-based population redistribution, and dasymetric simulation. The accuracy of the land cover data used in WorldPop data is low, and the spatial distribution of the population is inconsistent with that of land use in a small-scale space. To ensure the accuracy of the data, high-precision land-use data were used as the only auxiliary data, and the following method was proposed to facilitate the fine division of the population grid data:

(1) In the data processing stage, all used data were unified into the CGCS2000 coordinate system. The land-use data were summarized into 12 first-level categories according to the land-use classification standard of land-use change survey data, and WorldPop data were vectorized. The administrative boundaries, land use, and WorldPop data were superimposed and analyzed.

(2) In the population redistribution stage, land weight was established using the zoning method. The natural breakpoint was used to divide the population density into three density levels (high, medium, and low) to determine the land weight. Under the dual constraints of land and area weight, the WorldPop population was allocated to land in the 100 m grid.

(3) In the dasymetric simulation stage, based on the dasymetric method, 100 m × 100 m population redistribution grid data were used to allocate the population to the 25 m grid using the area weight method, and 25 m population grid data were obtained.

Stages (2) and (3) are the key stages of the proposed method and are introduced in detail subsequently.

### Population redistribution model

#### Land-type weight

In regions with large differences in population density, using a single model to explain the spatial distribution of the internal population is difficult. The zoning method can perform secondary zoning modeling of the study area according to the corresponding characteristics to effectively solve the drawbacks of a single model. Simultaneously, different land-use types affect the distribution of the population to varying degrees. Therefore, we divided the density level according to WorldPop population density; the land-type weights of the different population density partitions also differed. According to the natural breakpoint method, scholars [26, 30, 31] have divided the results of population spatialization into three density levels, high, medium, and low, and verified the accuracy of the different levels. The fitting accuracy was found to be higher at high-, medium-, and low-density levels. Therefore, according to the population in the 100 m WorldPop, the natural breakpoint method was used to divide the population into three levels, and the weights of the three levels were calculated. The land-type weight (*D*_*mj*_) at the grid and land-type levels were calculated using the following formulas:

$$D_{mij}=\frac{A_{\left(i,j\right)}}{\sum_{j=1}^{j}A_{\left(i,j\right)}},$$ (1)

$$D_{mj}=\frac{\sum_{i=1}^{i}D_{mij}}{\sum_{i=1}^{i}\sum_{j=1}^{j}D_{mij}},$$ (2)

where *D*_*mij*_ represents the land-type weight of grid *i* land type *j* in grading *m* (*m* has three levels—specifically, high, medium, and low), and *A*_(*i*,*j*)_ represents the area of grid *i* and land type *j*. The area of the grid should be allocated to the land type in the grid; therefore, it must be normalized. *D*_*mj*_ represents the normalized land-type weight of land type *j* in typification *m*.

#### Area weight

The spatial distribution of the population is not only related to land-use types but also closely related to the area of land types. When analyzing the spatial distribution of the population, the use of different types of land use and differences in their areas must be considered. Therefore, the area-weight method is commonly used for population spatial distribution models. The formula for calculating area weight was as follows:

$$WA_{\left(i,j\right)}=\frac{A_{\left(i,j\right)}}{\sum_{j=1}^{j}A_{\left(i,j\right)}},$$ (3)

where *WA*_(*i*,*j*)_ denotes the area ratio of land type *j* in the *i*^th^ grid.

#### Population redistribution

The area-weight method without auxiliary data can allocate a population but it cannot accurately describe the spatial distribution of the population. This study combined the area weight with the land-type weight, as a comprehensive weight, to improve the accuracy of the population spatial distribution. The population redistribution model was expressed as follows:

$$f_{(i,j)=}D_{mj}WA_{\left(i,j\right)},$$ (4)

$$F_{\left(i,j\right)}=\frac{f_{\left(i,j\right)}}{\sum_{j=1}^{j}f_{\left(i,j\right)}},$$ (5)

$$P_{\left(i,j\right)}=P_{i}F_{\left(i,j\right)},$$ (6)

where *f*_(*i*,*j*)_ represents the comprehensive weight of land type *j* in grid *i*, *F*_(*i*,*j*)_ represents the final weight of land type *j* in grid *i*, *P*_(*i*,*j*)_ denotes the redistributed population of grid *i*, and *P*_*i*_ denotes the WorldPop population of grid *i*.

### Dasymetric model

Wright [32] proposed a dasymetric method that has been widely used by several researchers [33, 34]. This method subdivides population distribution data into small areas using spatial variables. The advantage of this method is that it ensures that original and target grid populations remain unchanged. Therefore, to obtain a refined population distribution, the 100 m spatial resolution population grid data were increased to 25 m. Based on the 100 m population redistribution data, the 25 m grid population spatial data were generated using the dasymetric method. This formula is expressed as follows:

$$P_{I}=\sum_{j=1}^{j}P_{\left(i,j\right)}\times \frac{A_{\left(I,j\right)}}{A_{\left(i,j\right)}},$$ (7)

where *A*_(*I*,*j*)_ represents the area of type *j* of the 25 m grid *I*, *A*_(*i*,*j*)_ the area of type *j* of the 100 m grid *I*, and *P*_*I*_ the population of the 25 m grid *I*. If grid *I* is entirely water, *P*_*I*_ is 0, and the population in grid *I* is summarized as grid *i*.

### Evaluation metrics

The selected accuracy evaluation indices include the Standard Deviation (SD), Normalized Root Mean Square Error (%RMSE), and Coefficient of Determination (R^2^). The calculation formulas are presented in Eqs (8)–(10). The SD evaluates the dispersion of the data, %RMSE reflects the overall accuracy level of the model error, and R^2^ measures the goodness of fit of the estimated results. The equations used to calculate these metrics were as follows:

$$SD=\sqrt{\frac{1}{N}\sum_{a}^{N}\left(X_{a}-\bar{X}\right)},$$ (8)

$$\%RMSE=\sqrt{\frac{1}{N}\sum_{a}^{N}{\left(X_{a}-Y_{a}\right)}^{2}}/\frac{1}{N}\sum_{a}^{N}Y_{a},$$ (9)

$$R^{2}=1-\frac{\sum_{a}{\left(Y_{a}-X_{a}\right)}^{2}}{\sum_{a}{\left(Y_{a}-\bar{Y}\right)}^{2}},$$ (10)

where *X*_*a*_ is the estimated population of street *a*, *Y*_*a*_ is the census count of street *a*, $\bar{Y}$ is the average population of all streets, $\bar{X}$ is the average population of the model, and *N* is the total number of streets.

## Experiments and analysis

### Land-type weight analysis

**Fig 3:**
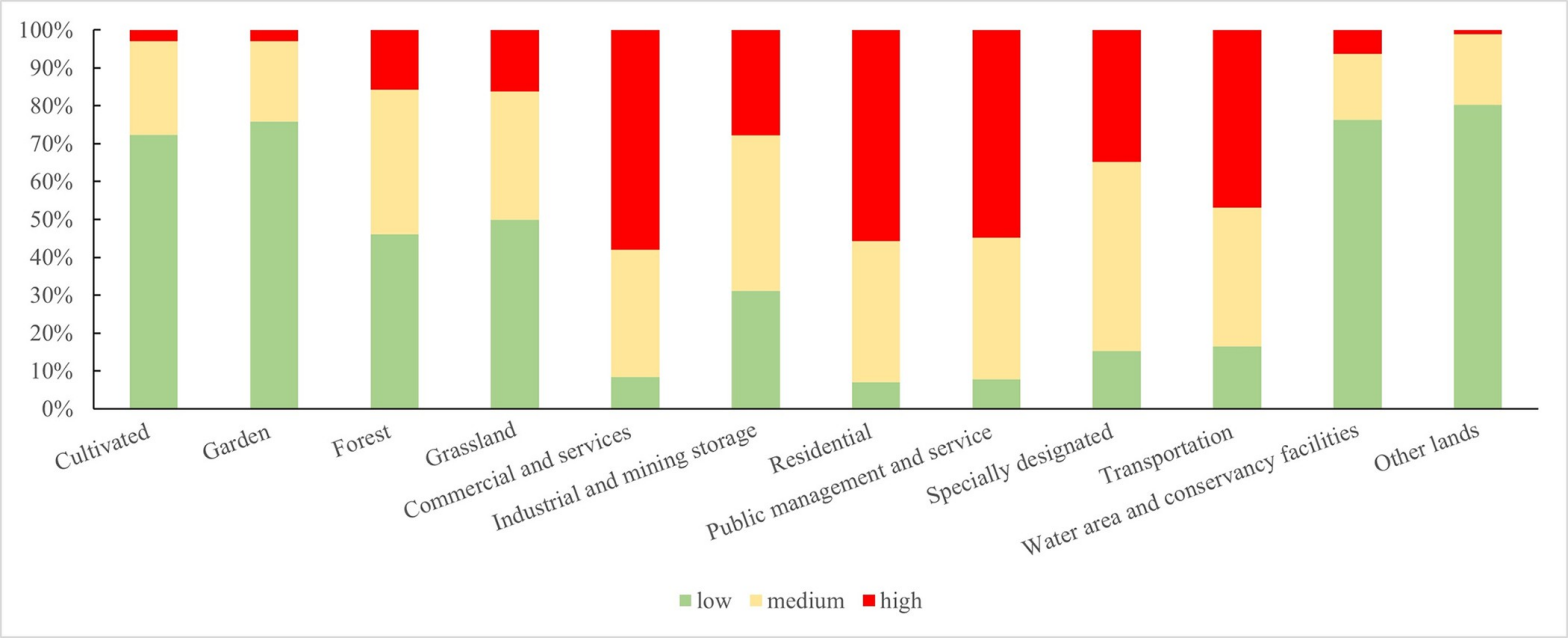
Partition land-type weight percentage accumulation map.

Using the above land weight method, the land weight values of the high, medium, and low levels were calculated. As illustrated in Fig 3, in the high population density area, the land-use types of residential, commercial and service, public management and service, and transportation land have the highest weights, and cultivated, garden, forest, grass, industrial and mining storage, water area, water conservancy facilities land, and other land types have the lowest, compared with the land-use types of medium and low population density levels. In the medium-population-density area, the land-use types of industrial and mining storage land and special land have the highest weight, compared with those of high- and low-population density levels. In the low-population-density area, the land weight of cultivated, garden, forest, grass, water area, water conservancy facilities, and other land was the highest, while that of commercial and service, residential, public management, and service, specially designated, and traffic land was the lowest, compared with the land-use types with high- and medium-population-density levels. This demonstrates that the method of calculating the weight of land type by partitioning aligns with the objective law of land-use type and population distribution.

Comprehensive analysis reveals that human activities are more frequent on residential land, commercial and service land, and other land types. By contrast, the intensity and frequency of human activity on cultivated, garden, forest, and other land types were relatively low. However, this does not imply that no human activity occurs on such land types; humans may engage in agriculture, horticulture, forestry, and other activities. Compared with other land types, the intensity and frequency of activities were lower; the weight value of the land types was smaller.

### Grid data accuracy analysis

Data from the seventh National Population Census were used as the evaluation standard, and the accuracy of the method was verified based on the following two aspects: First, the relative accuracy of the refined population spatial distribution data was evaluated by comparing the refined data with GPW, LandScan, and WorldPop, owing to the lack of effective calibration data within the 25 m grid. Second, because this method was refined, the degree of fit between the refined data and the WorldPop was verified.

#### Comparison with other data sets

**Fig 4:**
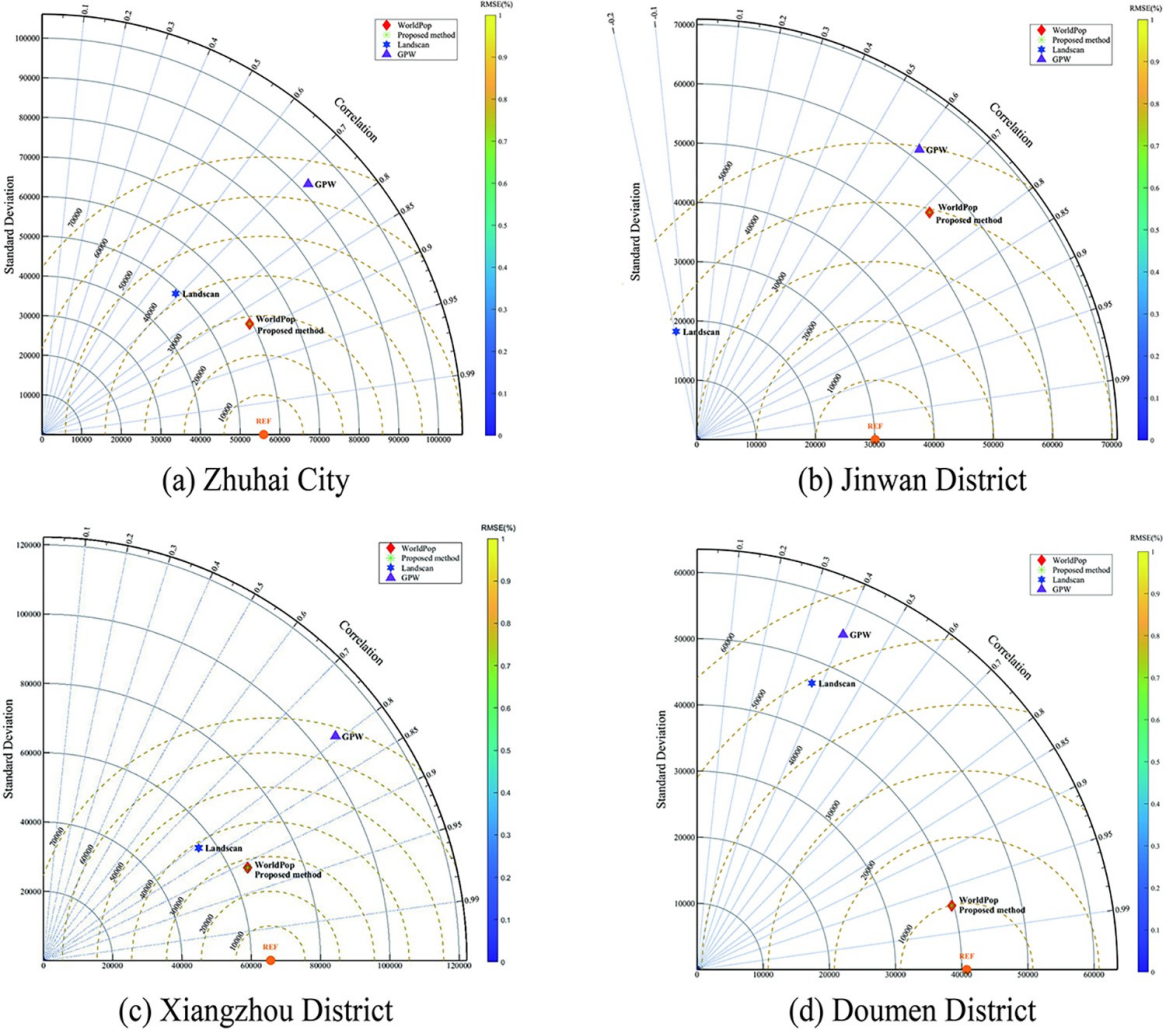
Taylor diagram of Zhuhai City and its districts and counties. Taylor diagram describing the overall accuracy of the different data, including WorldPop, GPW, LandScan, and study method.

A Taylor diagram was used to compare the accuracy of our results with the WorldPop, GPW, and Landscan datasets by summarizing the township, town, and sub-district categories to county-level administrative units. In Fig 4, the solid concentric arc represents the SD, the dotted concentric arc represents the %RMSE, and the angle between the line connecting the data point and the center of the circle with a radius of 90° represents R^2^. The closer the distance between the data point and the center of the dotted arc, the smaller the %RMSE. The smaller the distance between the data point and the center of the solid arc, the smaller the SD. The larger the angle of the fan formed by the connection between the data point and the center of the circle and the radius of 90°, the higher the R^2^. In other words, the smaller the distance between the data point and the REF point, the higher the accuracy, and the closer the distance is to the center of the solid arc, the higher the accuracy.

As illustrated in Fig 4(A), the method had a higher accuracy than the other three datasets in terms of SD, %RMSE, and R^2^ on the urban scale. To further analyze the difference between the results and those of the other datasets, the four datasets were compared on the county-level administrative unit. The results are presented in Fig 4(B)-4(D). In Fig 4(B), the SD and %RMSE of LandScan in Jinwan District were the smallest, but R^2^ exhibited overfitting. The results of this method were more accurate than those of the other three datasets in Xiangzhou and Doumen Districts. The accuracy advantage of this method was more evident in the Doumen District, which was much higher than that of the LandScan and GPW methods. This is because more water and water conservation facilities exist in this area, accounting for 52.3% of Zhuhai City, while the residential land is relatively less, accounting for only 27.0%. Employing land-use data to construct a population spatial distribution model can improve the accuracy of the region, indicating that the weight of the land type determined by this method can effectively identify the population of different land types. In general, in county-level administrative units, the results of this method are less accurate than those of WorldPop; however, the accuracy is higher than that of the other two datasets.

#### Fitting analysis with WorldPop

**Fig 5:**
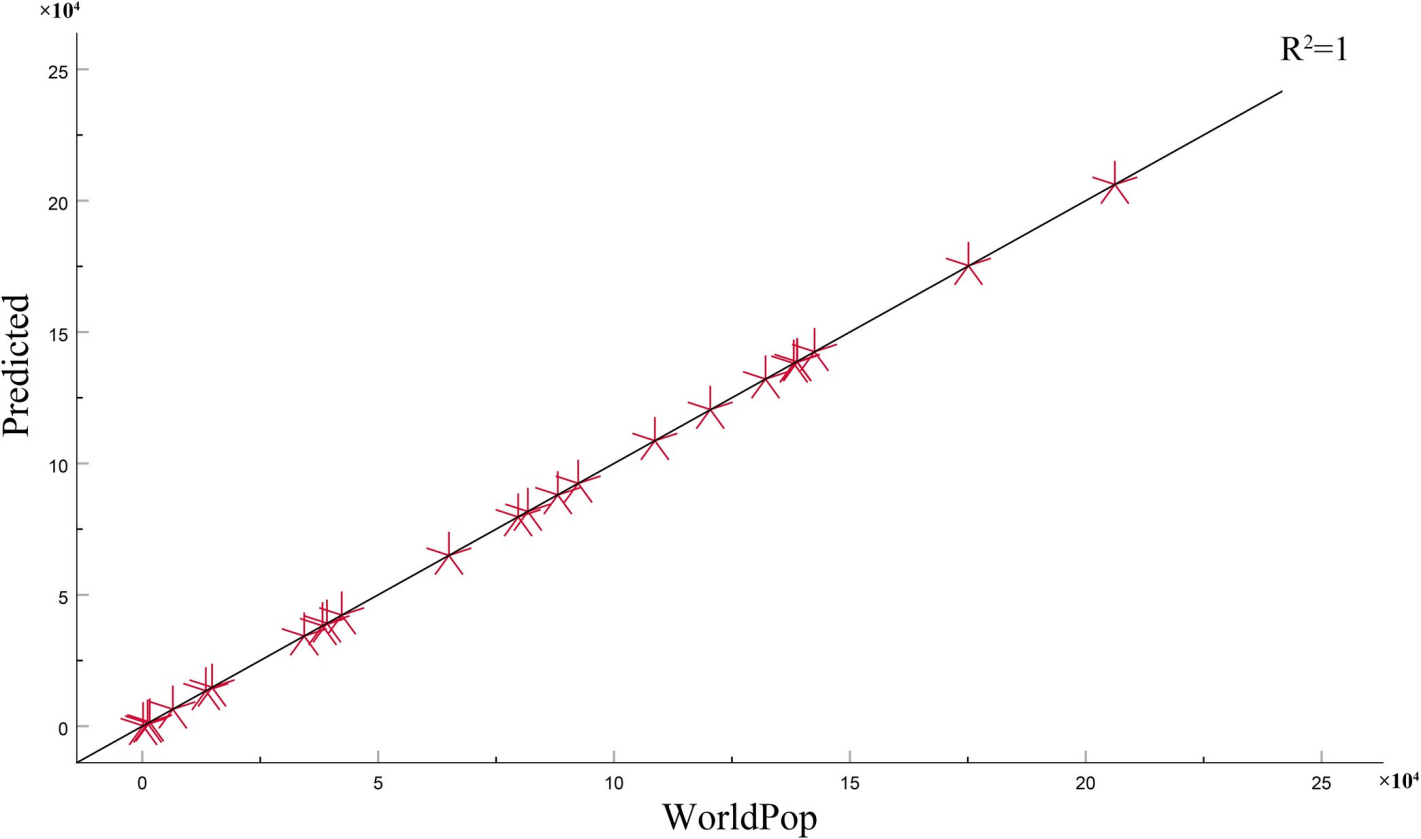
Accuracy comparison between the refined results and WorldPop. The abscissa represents the population of WorldPop, and the ordinate represents the population of the refinement result, in the township-level administrative unit scale.

The degree of fit of the refined grid data and WorldPop data of this method to the township, town, and Sub-district categories to the county-level administrative unit is illustrated in Fig 5. The difference between this method and WorldPop is that the land-use type data is added. By comparing the trend line R^2^ fitted by the two, the accuracy of this method is less different from that of WorldPop data. At the city-level scale, the refined results have a high degree of fit with the WorldPop data, and there is almost no deviation. The data accuracy improved slightly after the refinement of the township-scale analysis. The above accuracy verifies that the accuracy of the 25 m refined population grid data obtained by this method is more accurate.

## Discussion

**Fig 6:**
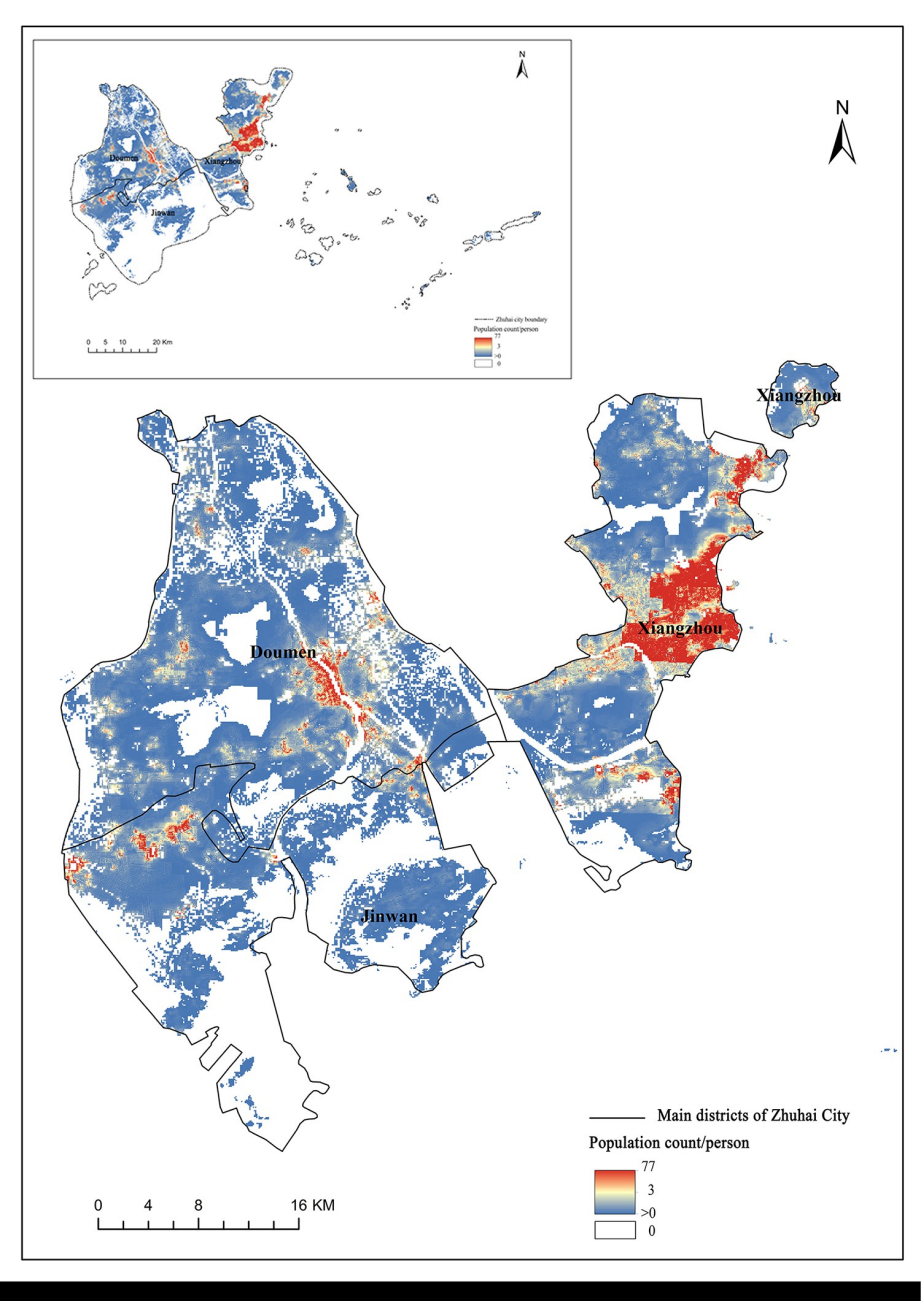
Refined population spatial distribution map of 25 m spatial resolution in Zhuhai City. The small map is the result map of Zhuhai City, and the large map is that of the main area.

A population refinement experiment with a 100 m grid scale was conducted in Zhuhai City, Guangdong Province, China. The results reveal that the SD, %RMSE, and R^2^ of the proposed method were lower than those of the WorldPop, GPW, and LandScan datasets, thereby effectively improving the accuracy of the population spatial distribution data. The refined results of the population spatial distribution at 25 m resolution in Zhuhai City are presented in Fig 6. From the perspective of spatial form, the spatial distribution of the population in Zhuhai City exhibits a decentralized concentric circular structure. For example, the population in Xiangzhou District gathered at the center and radiated out to the periphery, while the population distribution in Doumen and Jinwan Districts was more dispersed because of the large number of water areas, water conservancy facilities, and forest land.

**Fig 7:**
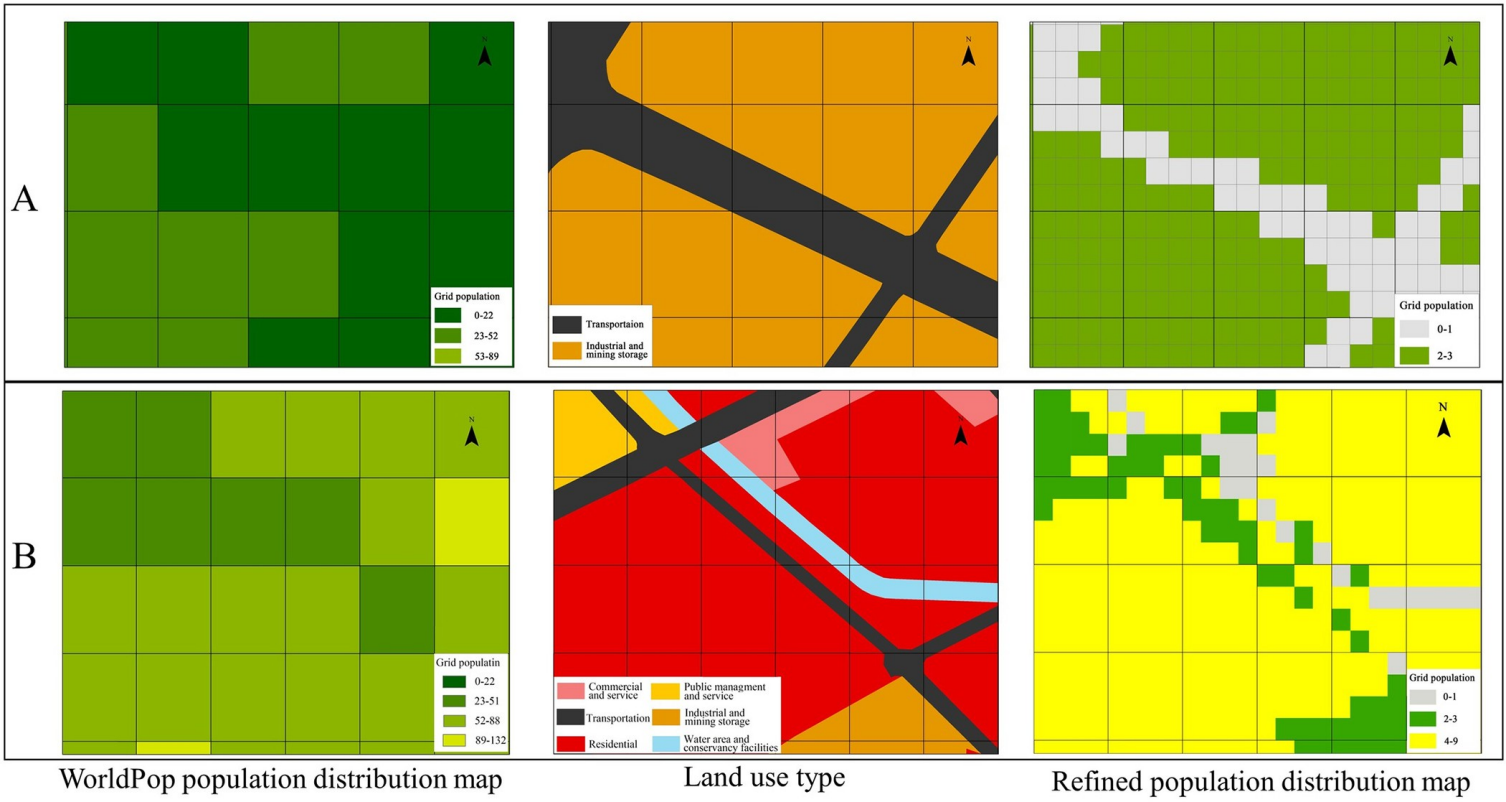
Comparison of population distribution within the grid. Two groups of visual grid population distribution comparison maps of A and B are listed. The land types presented in the land-use type map are the categories visible in the current map.

The differences in the population grids before and after refinement in Groups A and B are presented in Fig 7. The population distribution of WorldPop does not fully present the spatial distribution characteristics; however, after refinement, the population distribution better exhibits these characteristics of the population in the land-use type. The finely divided grid data accurately identified land types with fewer population activities—such as industrial and mining storage, water and water conservancy facilities, transportation, and other land types—and correspondingly reduced the population of these land types. Population is allocated to adjacent residential, commercial, and service lands. Compared with the WorldPop dataset, the population grid data obtained by this method exhibited a higher fit accuracy for the spatial accuracy of the population in the study area.

**Fig 8:**
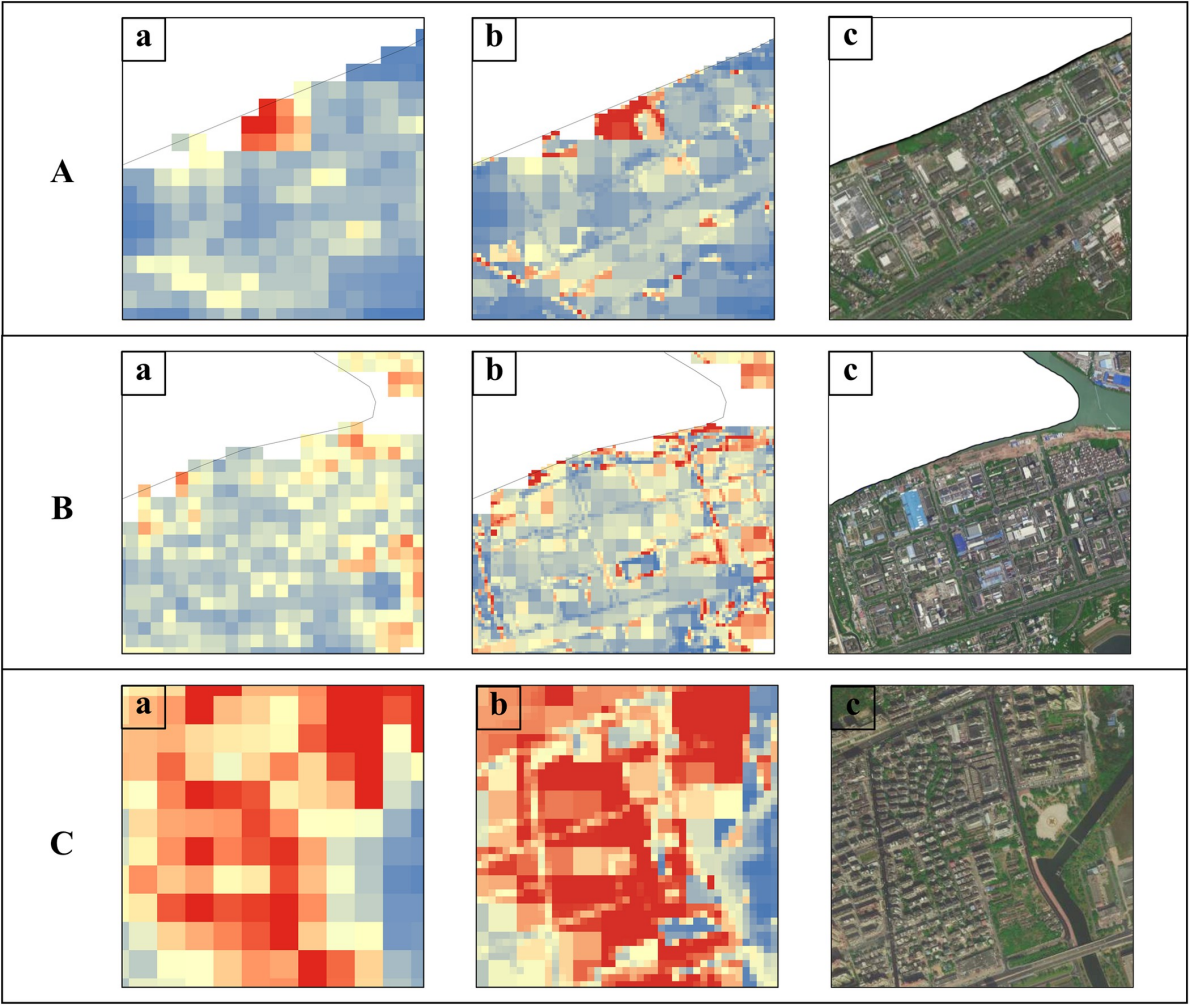
Local comparison: WorldPop, refinement results, and Amap. Three groups of visual comparison maps A, B, and C, where (a) represents WorldPop, (b) is the refined population distribution, and (c) is Amap from https://ditu.amap.com/.

Simultaneously, to further verify the effect of the model, we analyzed the local characteristics of WorldPop, refined data, and remote sensing image data. According to Fig 8, the refined data accurately identified land types with weak population activities, such as rivers and roads. Evident boundaries exist between land types with strong population activities, such as residential land. The results indicate that the refinement reflects the consistency of population spatial distribution and land-use unit distribution and that the population density in the refined data is more aligned with the actual situation. The refined data of population spatial distribution can better reflect the detailed characteristics of such a distribution, while retaining the macro-distribution characteristics of the population—such as depicting the detailed characteristics of traffic, water systems, housing, and other land types—and better depicting the internal differences of the region.

The refinement results are often affected by the auxiliary data’s accuracy. In general, the use of auxiliary data with lower accuracy can relatively improve the refinement results, but some detailed information will be lost; the use of high-precision auxiliary data can reveal more detailed information, but the acquisition of such data may be subject to some restrictions. Therefore, the uncertainty of this method derives from the selection of auxiliary data. To obtain more detailed population spatial distribution data, this study selected WorldPop data and land-use data with higher accuracy and resolution as auxiliary data. In theory, this method is also applicable to other precision auxiliary data. In practical applications, the actual situation must be considered to select appropriate auxiliary data for refined research.

## Conclusions and future work

This study proposed a partition refinement method for the spatial distribution data of the WorldPop population. This method has simple steps, requires a limited amount of data processing, and is not constrained by the scope of the study area, thereby overcoming the dependence on demographic data inherent in previous research on the spatial distribution of the population. In addition, this method not only achieves refined population spatial distribution and simulation and improves the spatial resolution of population spatial distribution but also effectively utilizes and improves the spatial distribution data of the public population. Practically, this study can effectively solve the problem of public population spatial distribution data.

The proposed method contributes to the research on population spatial distribution refinement; however, deficiencies still need to be addressed. In future research, the problems between adjacent grids must be considered and corrected. In addition, we can try combining multi-source auxiliary data with existing products by analyzing the characteristics of different population-space products to construct high-precision and high-efficiency refined models and methods. A future research direction can involve comprehensively utilizing the characteristics of different auxiliary data, mining more accurate and effective social and economic data, and conducting high-precision spatiotemporal dynamic analyses.
